# Data to model the influence of CSR on consumer behaviors: A process approach

**DOI:** 10.1016/j.dib.2019.104713

**Published:** 2019-10-23

**Authors:** S. Castro-González, B. Bande, P. Fernández-Ferrín, T. Kimura

**Affiliations:** a Universidade de Santiago de Compostela, Spain; b University of the Basque Country UPV/EHU, Spain; c Hosei University, Japan

**Keywords:** Corporate social responsibility (CSR), Consumer behavior, Process procedure

## Abstract

The aim of this research is to present inferential statistical data on the influence of Corporate Social Responsibility (CSR) perceptions on consumer advocacy behaviors, and to consider when and how that relationship occurs. Data was provided by 252 customers of a food company located in Spain. Structural equation modeling was used to analyze the psychometric proprieties of the measurement scales and to test the proposed direct hypotheses; conditional process analysis was used to test the proposed mediation hypothesis. The data indicate that CSR practices positively influence consumer advocacy behaviors through consumer admiration – the higher the integrity, the stronger the effect. This article provides data related to “*Corporate social responsibility and consumer advocacy behaviors: The importance of emotions and moral virtues”* [1].

**Table:** Specifications Table

Subject area	*Marketing*
More specific subject area	*Consumer behavior, CSR and consumers*
Type of data	*Table, graph and figure*
How data was acquired	*Data was collected through questionnaires from customers. Supplementary file contains the questionnaire.*
Data format	*Raw, analyzed, descriptive and statistical data*
Experimental factors	*Sample is composed of customers of a food company located in Spain. A research agency, which name is Instituto Sondaxe S.L., collected the data using the proposed questionnaire. In addition to the variables of the proposed model, the questionnaire contained demographic variables and a control question*
Experimental features	*CSR perception and admiration are instruments to influence advocacy behaviors. Integrity moderates those relationships*
Data source location	*Ourense, Galicia, Spain*
Data accessibility	*Data is included in this article*
Related research article	*S. Castro-González, B. Bande, P. Fernández-Ferrín, T. Kimura, Corporate social responsibility and consumer advocacy behaviors: The importance of emotions and moral virtues, J. Clean. Prod. 231 (2019) 846–855.*https://doi.org/10.1016/J.JCLEPRO.2019.05.238 [1]

**Table:** 

**Value of the Data** • This data presents a way to contrast how CSR practices influence on consumer behaviors, which can guide other researchers toward designing similar models for expanding investigations in this context. • Data can be used as a springboard to contrast alternative models with these variables. They shall, therefore, facilitate further studies on this field of research. • Data within this article can be used by researchers as comparison materials with other data obtained from other industries, cities, regions, or countries. • For future researches, academics can use the questionnaire. It is a validated tool, so they only have to apply it to their data. • The method employed in this study could be extended towards other organizational researches. The process is displayed on the flow diagram of overall methods.

## 1 Data

**Fig. 1:**
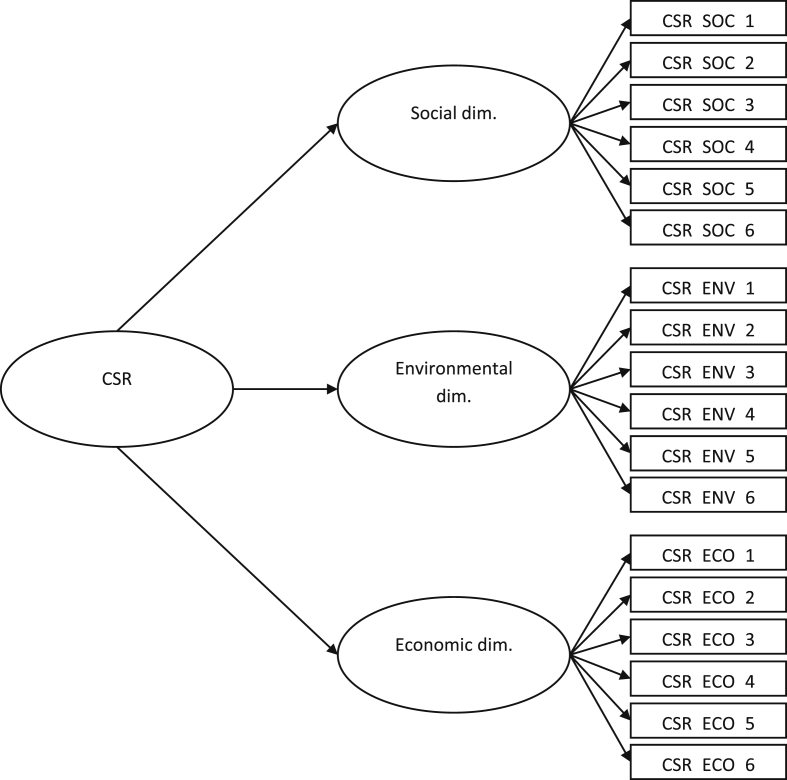
Measurement model of CSR.

**Fig. 2:**
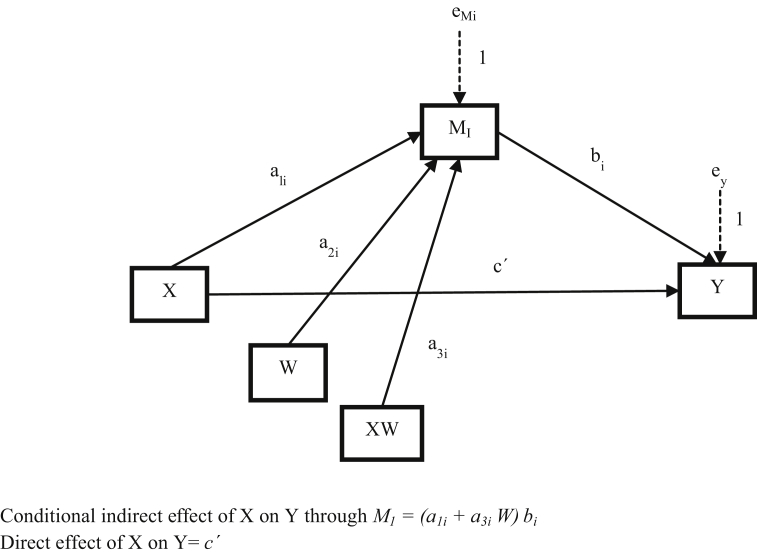
Statistical diagram of model 7 of the Process for SPSS. Source: Hayes [6].

The dataset in this article describes the method to contrast how CSR influence on customers' behaviors. Table 1 describes the demographic and sociological characterization of the sample. Table 2 shows the variables, items codes, and the values to evaluate scales reliability and validity. Table 3 shows the main coefficients for the conditional process analysis. Fig. 1 schematizes how to measure CSR as a second-order construct. Fig. 2 describes how relationships proposed in the model are probe through model 7 of the Process for SPSS. Supplementary file, on the one hand, contains the questionnaire used for data collection; on the other hand, holds the flow diagram of overall methods from the data collection up to the analysis performed.

**Table 1:** Classification of respondents by gender, age and educational qualification.

	Frequency	Percentage
**Gender**		
Male	107	42.5%
Female	145	57.5%
	252	100%
**Age**		
18–34	79	31.3%
35–54	92	36.5%
54–Above	81	32.1%
	252	100%
**Educational qualification**		
Without studies	4	1.6%
Primary studies	53	21.0%
Secondary studies	45	17.9%
Tertiary type A	109	43.2%
Tertiary type B	23	9.1%
Master's degree	7	2.8%
No answer	11	4.4%
	252	100%

**Table 2:** Variables, measures, codes and scales reliability and validity.

Construct		CODES	Mean	SD	Factor loadings	Cronbach's alpha	Composite reliability	Average variance extracted (AVE)
CSR	CSR Social dimension	CSR_SOC_1	4.58	1.85	0.90	0.95	0.95	0.76
		CSR_SOC_2	4.45	1.89	0.90			
		CSR_SOC_3	4.63	1.89	0.74			
		CSR_SOC_4	4.58	1.91	0.95			
		CSR_SOC_5	4.77	1.89	0.90			
		CSR_SOC_6	4.76	1.81	0.82			
	CSR Economic dimension	CSR_ECO_1	6.00	1.25	0.56	0.88	0.85	0.50
		CSR_ECO_2	5.60	1.45	0.84			
		CSR_ECO_3	5.35	1.55	0.87			
		CSR_ECO_4	5.27	1.49	0.74			
		CSR_ECO_5	6.08	1.24	0.57			
		CSR_ECO_6	6.12	1.27	0.58			
	CSR Environmental dimension	CSR_ENV_1	4.23	1.87	0.86	0.96	0.94	0.72
		CSR_ENV_2	4.26	1.70	0.93			
		CSR_ENV_3	4.15	1.75	0.66			
		CSR_ENV_4	4.11	1.83	0.97			
		CSR_ENV_5	4.58	1.66	0.82			
		CSR_ENV_6	4.43	1.73	0.79			
Admiration		ADM_1	4.37	1.90	0.81	0.93	0.92	0.71
		ADM_2	5.04	1.74	0.66			
		ADM_3	3.01	1.96	0.88			
		ADM_4	3.27	1.96	0.93			
		ADM_5	3.00	1.96	0.89			
Advocacy behaviors		ADV_B_1	5.89	1.49	0.73	0.86	0.87	0.63
		ADV_B_2	4.98	1.91	0.91			
		ADV_B_3	5.10	1.97	0.75			
		ADV_B_4	4.52	2.27	0.76			
Integrity		INTEGRI_1	6.58	0.78	0.61	0.73	0.77	0.63
		INTEGRI_2	6.54	0.74	0.95

**Table 3:** Model coefficients summary for the conditional process analysis.

Relationship	Coeff.	p
*a*_*1*_	0.600	<.001
*b*_*1*_	0.455	<.001
*a*_*3*_	0.232	0.006

## 2 Experimental design, materials, and methods

### 2.1 Data characterization

The research adopted a survey research design to obtain data from 252 customers of a Galician food company in Spain through a structured questionnaire which employed 7-point Likert scales and control questions. The questionnaire, which provides information on how the variables were measured, can be consulted in the Supplementary file.

The data shows that (i) the majority of the respondents were female (57.5%); (ii) the respondents' age groups were of a similar size, although the 35–54 age group (36.5%) is slightly higher; and (iii) most of the respondents were in the tertiary type A educational group (43.2%). Table 1 summarizes the descriptive analysis.

The contrasted model includes four dependent and independent variables (CSR, admiration, advocacy behaviors, and integrity) and two control variables (gender and level of education). It is important to note that CSR has been considered as a second-order construct [2]. That is, CSR is a latent construct, which includes three sub-constructs or dimensions, specifically, the social dimension, the environmental dimension, and economic dimension. The social dimension tries to capture whether the company is trying to promote educational, cultural or public health programs, among other aspects. The environmental dimension is associated with the desire to protect the environment and make appropriate use of resources, avoiding wasting them. Finally, economic dimension refers to the company's search for higher quality products, higher productivity, or better financial performance. Fig. 1 represents how to use that variable in the model.

To examine the dataset and to contrast the hypotheses, a combination of tools has been used – specifically SPSS, AMOS 24 and Process Procedure for SPSS.

### 2.2 Psychometric proprieties of the measurement scales

To make sure that the questionnaire captures what it is assumed to measure, the content reliability and validity methods were used. The scale's internal reliability was assessed in two ways: through the analysis of Cronbach's alpha (α) [3] and through the composite reliability indices (IFC) [4]. Table 2 contains this information.

To examine the validity of the measurement model, a confirmatory factor analysis (CFA) was performed. The model indices obtained through AMOS 24 were: X^2^ measure, root mean square error of approximation (RMSEA), comparative fit index (CFI), incremental fit index (IFI), Tucker–Lewis index (TLI). To assess discriminant validity, Fornell and Larcker's [5] approach was used.

### 2.3 Hypothesis testing

The hypotheses formulated for the research were (see proposed model on [1]):

H_1_: CSR is positively related to a consumer's admiration for a company.

H_2_: Admiration is positively related to advocacy behaviors.

H_3_: Admiration mediates the relationship between CSR and advocacy behaviors.

H_4_: Consumer integrity moderates the influence of CSR on admiration, such that the relationship is stronger for consumers with high integrity and weaker for consumers with low integrity.

To test the proposed hypotheses (some of which involve mediating and moderating effects) a conditional process analysis was conducted – specifically, using Hayes' [6] model 7 of the PROCESS Procedure for SPSS Release 2.16. Fig. 2 shows the statistical diagram of the model where X was the CSR variable, M was admiration, Y was advocacy behaviors, and W was integrity.

To avoid interpretation problems with some of the coefficients, the CSR and integrity variables, which are those involved in the interaction terms, were mean centered. To test the interaction and to make statistical inferences about the conditional effects, the pick-a-point method [7] combined with bootstrapping was used. The results of these analyses appear in the research article related to this work [1]. A summary of the results specifically related to the proposed model is presented in Table 3.
