# Supplementary material for: Data to model the influence of CSR on consumer behaviors: A process approach
Source: Data Brief. 2019 Oct 23;27:104713. doi: 10.1016/j.dib.2019.104713 (PMC6849069; doi:10.1016/j.dib.2019.104713)

SUPPLEMENTARY FILE

S.1 Survey used in the study.

Do you know company *[Company name]*?

***CSR Social dimension**

In my opinion, regarding society, *[Company name]* is really…

… Trying to sponsor educational programmes

… Trying to sponsor public health programmes

… Trying to be highly committed to well-defined ethical principles

… Trying to sponsor cultural programmes

… Trying to make financial donations to social causes

… Trying to help to improve quality of life in the local community

***CSR Environmental dimension**

In my opinion, regarding the environment, *[Company name]* is really…

… Trying to sponsor pro-environmental programmes

… Trying to allocate resources to offer services compatible with the environment

… Trying to carry out programmes to reduce pollution

… Trying to protect the environment

… Trying to recycle its waste materials properly

… Trying to use only the necessary natural resources

***CSR Economic dimension**

In my opinion, regarding the economy, *[Company name]* is really…

… Trying to maximise profits in order to guarantee its continuity

… Trying to build solid relations with its customers to assure its long-term economic success

… Trying to continuously improve the quality of the services that they offer

… Trying to have a competitive pricing policy

… Trying to always improve its financial performance

… Trying to do its best to be more productive

***Admiration**

I feel admiration when I think about *[Company name]*

I feel respect when I think about *[Company name]*

I feel inspired when I think about *[Company name]*

[Company name] amazes me

[Company name] inspires me

***Advocacy Behaviors**

To try new products introduced by *[Company name]*

To blog in favor of *[Company name]*

To give another chance to *[Company name]*, if it does something that you don't like

To provide helpful feedback to *[Company name]*

***Integrity**

I conduct myself by the same values that I talk about

When I promise something, you can be certain that it will happen

Age

Gender
Educational Level

Without studies
Primary studies
Secondary studies
Tertiary type A
Tertiary type B
Master’s degree

** Likert scales (1=totally disagree; 7=totally agree)*

Figure S.1. Flow diagram of the overall methods of the study

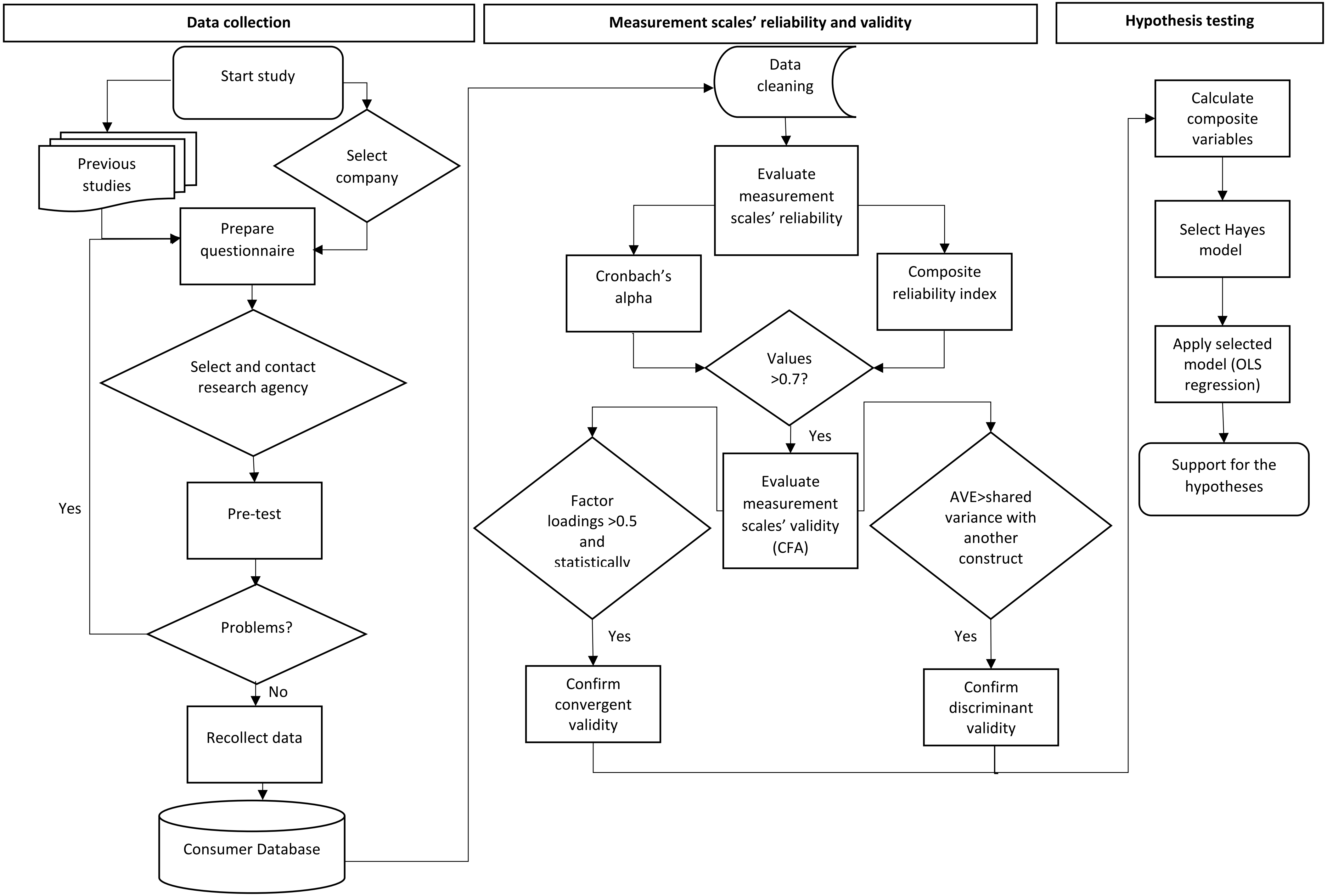

Supplement: Multimedia component 2 [file mmc2.pdf]
